# Chickpea (*Cicer arietinum* L.): Integrating Nutritional Excellence, Health Benefits, and Abiotic Stress Resilience for Sustainable Food Systems

**DOI:** 10.3390/foods15111982

**Published:** 2026-06-03

**Authors:** Ting Luo, Tong Wu, Kexin Liu, Yifan Li, Jinyao Li, Weilan Wang

**Affiliations:** Xinjiang Key Laboratory of Biological Resources and Genetic Engineering, College of Life Science & Technology, Xinjiang University, Urumqi 830046, China; lt18160225400@163.com (T.L.); 17609071576@163.com (T.W.); liukexin990528@163.com (K.L.); 13669242279@163.com (Y.L.)

**Keywords:** chickpea, nutritional components, biological activity, abiotic stress, breeding

## Abstract

Chickpea (*Cicer arietinum* L.) is a major annual legume crop with a balanced nutritional profile and a broad spectrum of bioactive constituents; these characteristics have made it a useful ingredient in health-oriented food applications. Chickpea supplies protein that is readily absorbed and digested, along with isoflavones and other bioactive plant compounds that act on physiological pathways associated with chronic disease prevention. Nonetheless, the combined pressures of drought, heat, cold, and salinity persistently limit its yield potential and cultivation stability. This review integrates the most recent progress in chickpea research, with emphasis on its intrinsic value derived from macronutrients, micronutrients, and bioactive metabolites. It further synthesizes the physiological determinants and metabolic reprogramming mechanisms underlying abiotic stress tolerance, outlines precision breeding strategies for developing resilient and high-quality ideotypes, and examines pathways for the high-value utilization of chickpea-derived processing by-products. Future efforts should focus on developing stress-resilient cultivars and expanding chickpea’s application in functional food innovation.

## 1. Introduction

**Figure 1:**
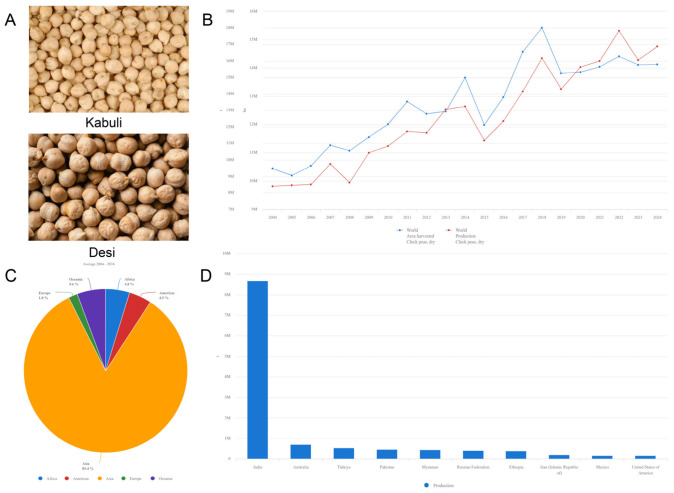
(**A**) Kabuli and Desi. (**B**) Production/Yield quantities of chickpeas, dry in World+ (Total) from 2004 to 2024. (**C**) Share of different production by region in total pulse production. (**D**) Top ten chickpea-producing countries in the world.

Chickpea (*Cicer arietinum* L.), also known as Bengal gram, is an annual legume species traditionally classified into two market types based on seed morphology: the light-colored, larger, and smooth-seeded Kabuli type, and the smaller, dark-seeded Desi type with a thicker seed coat (Figure 1A) [1]. As a globally important food and economic crop, chickpea is predominantly cultivated in temperate and semi-arid regions, where its production is closely shaped by sown area and regional agronomic conditions [2]. Long-term agricultural records from 2004 to 2024 show steady increases in chickpea production and harvested area during a period marked by worldwide initiatives targeting hunger and malnutrition, with Figure 1B depicting this upward trajectory (FAOSTAT. Available online: https://www.fao.org/faostat/en/#data/QCL/visualize [accessed on 17 May 2026]). Figure 1C further highlights the regional distribution, with Asia accounting for 83.4% of global output in 2024, followed by Oceania, Africa, the Americas, and Europe. In China, Mulei County of Xinjiang represents the principal production zone, contributing over 80% of national output through a consolidated industrial chain. On a global scale, India remains the largest producer, with Australia, Turkey, and Pakistan representing additional major contributors (Figure 1D).

Chickpea’s widespread acceptance across cultures stems from its rich nutrient composition and diverse array of bioactive compounds. It contains substantial levels of carbohydrates, high-quality protein, unsaturated fatty acids, polyphenols, dietary fiber, minerals, and vitamins, making it a nutritionally dense food often referred to as the “poor man’s meat” [3]. Its protein profile is distinguished by the presence of eight essential amino acids, with leucine (8.7%), arginine (8.3%), and lysine (7.2%) representing the major contributors [4]. Moreover, chickpea seeds contain numerous isoflavones—including biochanin A, maackiain derivatives, formononetin, and genistein—that exhibit antioxidant, antifungal, and reproductive regulatory activities [5]. To date, more than 1600 isoflavone structures have been characterized in legume species [6]. Modern dietary patterns, characterized by elevated intake of saturated fats and insufficient dietary fiber, have contributed to rising incidence of hypertension, dyslipidemia, and diabetes. In addition to pharmacological interventions, dietary modification is recognized as an essential complementary strategy. Legumes, particularly chickpea, offer functional components with demonstrated antioxidant, hypoglycemic, lipid-modulating, anti-inflammatory, and antimicrobial potential [7,8].

Global climate change poses mounting threats to agricultural productivity, with drought, extreme temperature, salinity, and heavy metal contamination representing the most consequential abiotic stressors for crop systems [9]. These stresses initiate complex cascades of metabolic and physiological disruption, undermining cellular homeostasis and impairing key biochemical processes [10]. Enhancing chickpea resilience therefore requires a mechanistic understanding of stress perception, signal transduction, and adaptive responses across developmental stages [11]. This review synthesizes current advances in chickpea research, emphasizing its nutritional composition, bioactive constituents, and major abiotic constraints. Building on previous findings, we evaluate chickpea’s genetic improvement potential, discuss associated trade-offs, and explore high-value utilization of processing by-products. Our aim is to provide an integrated reference for advancing chickpea-based food innovation, strengthening breeding strategies for stress tolerance, and expanding its role in sustainable agriculture.

## 2. Nutritional Composition

Chickpea contains a broad spectrum of nutrients and bioactive constituents, with its major nutritional categories, associated phytochemicals, and their distribution summarized in Table 1.

### 2.1. Protein

Chickpea is a high-quality source of plant protein, with a crude protein content that typically accounts for 21.07% of its dry weight. Its protein fractions primarily consist of globulin (53–60%, of which nearly 97% is legumin), glutelin (19–25%), albumin (8–12%), and prolamin (3–7%) [18]. Protein extracts derived from chickpea frequently contain peptides with molecular weights ranging from 0.3 to 11 kDa (approximately 3–11 amino acids) [8]. In terms of amino acid composition, chickpea is rich in aspartic acid and glutamic acid, alongside essential amino acids such as leucine and arginine. Although its content of sulfur-containing amino acids (e.g., methionine and cysteine) is relatively limited, this deficiency can be offset through dietary complementation with cereals. Furthermore, compared with soybean protein isolates, chickpea proteins demonstrate superior bioavailability and in vitro digestibility (37–52%).

The physicochemical properties of chickpea proteins are significantly influenced by genotype. Typically, the dark-seeded Desi types exhibit a slightly higher total crude protein content, whereas the light-seeded Kabuli types display superior functional properties, including a higher protein solubility index and better in vitro digestibility. Processing techniques can markedly alter the spatial conformation of macromolecules and influence protein quality. Among processing methods, industrial extrusion and domestic dry roasting are more effective at preserving protein quality than conventional boiling [19]. Traditional soaking results in only minor losses of water-soluble albumin and free amino acids, whereas subsequent thermal treatments (such as steaming or microwaving) induce the thermal denaturation and structural unfolding of macromolecular globulin. These physical modifications not only completely inactivate heat-labile antinutritional factors, such as trypsin inhibitors, but also substantially enhance the in vitro protein digestibility of the final cooked products.

### 2.2. Carbohydrates

Carbohydrates constitute the principal component of chickpea, representing approximately 60–65% of its dry weight [3]. The digestible fraction primarily includes free sugars and starch (accounting for 30.8–37.9% of its dry mass), whereas the nondigestible fraction is predominantly composed of dietary fiber, including insoluble fiber (27.84%) and soluble fiber (1.42%) [3,20].

The carbohydrate profile is significantly influenced by genotype. The dark-seeded Desi types possess a thicker and highly lignified seed coat, resulting in a substantially higher total content of insoluble dietary fiber (e.g., cellulose and hemicellulose) compared with the Kabuli types. Conversely, the light-seeded Kabuli types tend to accumulate higher proportions of total starch and free soluble sugars (e.g., fructose and sucrose) within their inner cotyledons. Processing methods also markedly alter carbohydrate composition. Soaking and boiling lead to the substantial leaching of oligosaccharides (with reductions exceeding 60%). Furthermore, pressure or conventional cooking induces the irreversible gelatinization of starch, while subsequent amylose retrogradation during cooling and storage increases the proportion of resistant starch.

### 2.3. Lipids

Although lipids represent a relatively small fraction of chickpea’s total nutritional profile (2.70–6.48%), their caloric contribution is more than twice that of an equivalent amount of protein or carbohydrate [21]. The fatty acid spectrum is dominated by nutritionally important polyunsaturated and monounsaturated fatty acids. Linoleic acid (C18:2, accounting for 51.2% of total fatty acids) and oleic acid (C18:1, accounting for 32.6%) constitute the primary components [3]. Additionally, it contains minor components such as phytosterols and tocopherols, which directly influence the oxidative stability of the products, as well as the textural characteristics, flavor profile, and shelf life of chickpea-based products [22].

Among genotypes, the light-seeded Kabuli types tend to accumulate higher levels of monounsaturated oleic acid, whereas the dark-seeded Desi types contain a greater proportion of polyunsaturated linoleic acid. This distinct fatty acid profile confers upon the Kabuli types superior natural oxidative stability compared with the Desi types. Regarding processing, because lipids are typically stably encapsulated within the matrix network of cotyledon cells, conventional soaking exerts a minor effect; however, dry-heat treatments (such as roasting or frying) can readily induce the mild degradation or isomerization of polyunsaturated fatty acids due to thermal oxidation.

### 2.4. Minerals and Vitamins

Chickpea is rich in potassium, magnesium, iron, zinc, copper, phosphorus, and manganese, serving as an important dietary source of various essential trace elements [23]. The levels of these elements are strongly influenced by environmental conditions and exhibit relatively modest genotype effects, often complicating quantitative assessment [13]. With respect to vitamins, chickpea provides both fat-soluble and water-soluble forms, including retinol, vitamin C, thiamine (B1), vitamin E, and folate. Folate acts as a coenzyme in one-carbon transfer reactions and plays a central role in cellular metabolism. Chickpea also contains additional B-complex vitamins—B2, B5, and B6—with measurable varietal differences. For example, Desi chickpeas contain 0.21, 1.01, and 0.30 mg/100 g of these vitamins, respectively, compared with 0.26, 1.02, and 0.38 mg/100 g in Kabuli types [24].

Furthermore, Desi types are rich in anthocyanins, which are almost completely absent in Kabuli types. Processing methods also exert a profound impact: dehulling causes Desi types to lose the vast majority of polyphenols distributed in the seed coat. Soaking and high-temperature cooking lead to the substantial leaching and loss of water-soluble vitamins (such as folate) and free phenolic acids. Conversely, the germination process markedly enhances the lipid-soluble vitamin content in chickpea seedlings, thereby increasing its nutritional value as a plant-based food resource [25].

### 2.5. Polyphenols

#### 2.5.1. Isoflavones

**Figure 2:**
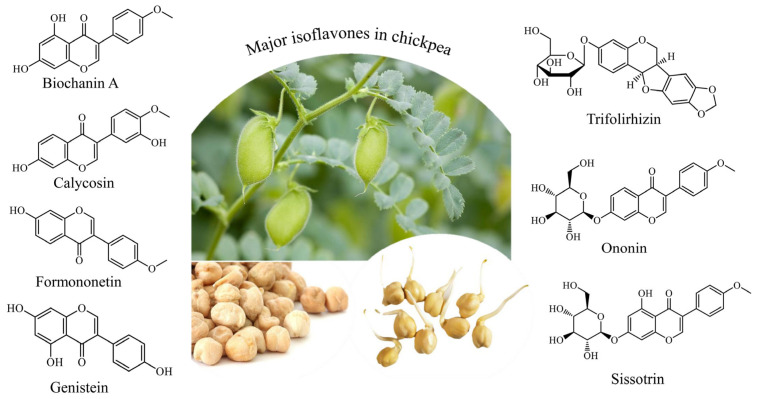
The molecular structure of the major isoflavones in chickpea.

The chickpea matrix is exceptionally rich in secondary phenolic metabolites, primarily including phenolic acids and flavonoids. Notably, chickpea is one of the very few non-soybean plants in nature that abundantly accumulates isoflavones, with a total isoflavone content of approximately 153–340 mg/100 g. Studies have identified seven isoflavones (Figure 2), among which biochanin A (0.18 ± 0.02 mg/10 g) and formononetin (0.10 ± 0.01 mg/10 g) are the absolute predominant components [26,27].

The polyphenol profile is significantly regulated by genotype. The light-seeded Kabuli types mainly contain pinocembrin, quercetin, kaempferol, and biochanin A derivatives. In contrast, the dark-seeded Desi types specifically synthesize large amounts of anthocyanins and condensed tannins (proanthocyanidins), making them a “hyper-accumulation zone” for polyphenols, with total phenolic and flavonoid contents reaching 5 to 13 times those of the Kabuli types [1,28]. Industrial dehulling causes Desi types to lose the vast majority of their seed coat polyphenols; although high-temperature cooking degrades heat-labile free phenolics, cell wall disruption promotes the release of insoluble bound phenolics. Bioprocessing interventions are even more drastic: under specific germination conditions (e.g., 33 °C, 171 h), the total flavonoid content of black chickpea can surge by nearly 115-fold [26,29]. Furthermore, germination and fermentation strongly activate endogenous β-glucosidase, promoting the substantial hydrolysis of higher-molecular-weight isoflavone glycosides into highly chemically reactive free aglycones.

#### 2.5.2. Saponins

Saponins are amphiphilic macromolecules composed of a hydrophobic triterpenoid backbone and hydrophilic sugar chains. The saponins in chickpea belong almost exclusively to the oleanane-type triterpenoid Group B soyasaponins, with a total content ranging approximately from 453 to 2072 μg/g. Mass spectrometric analysis indicates that saponin B and DDMP-type saponins are its most predominant chemical components [30]. Regarding tissue distribution, in stark contrast to polyphenols, which are extremely concentrated in the seed coat, saponin macromolecules are almost entirely distributed within the inner cotyledons and hypocotyl. Consequently, the Kabuli and Desi types do not exhibit substantial differences at the baseline total saponin concentration. Considering that Kabuli types typically possess a larger mass proportion of cotyledons, their absolute saponin extraction yield on a whole-seed basis is generally comparable to or even slightly higher than that of the Desi types.

During preparation, food processing interventions lead to varying degrees of saponin loss [31]. Because the triterpenoid chemical backbone of saponins exhibits excellent thermal stability, conventional microwaving, dry roasting, or short-term pressure cooking induces almost no significant degradation of its backbone. However, owing to their strong surface activity and water solubility, during prolonged water soaking prior to cooking and the subsequent boiling process, saponins readily form micellar complexes in the aqueous phase and undergo substantial leaching, thereby resulting in irreversible physical loss.

## 3. Biological Activities

**Figure 3:**
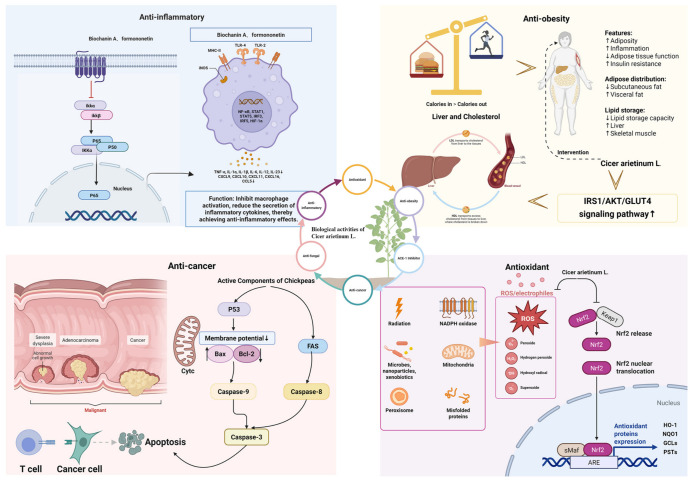
Principal biological activities of chickpea. Arrows indicate activation. T-shaped lines (⊣) represent inhibition.

Legumes have served as staple foods for several millennia, and in recent decades their health-promoting properties have received renewed scientific attention. Extensive evidence indicates that chickpea contains a wide array of bioactive compounds and demonstrates multiple physiological functions, including antioxidant activity, metabolic regulation such as cholesterol-lowering, hypoglycemic and hypolipidemic effects, inhibition of angiotensin-converting enzyme (ACE), anticancer activity, antifungal effects, and anti-inflammatory activity. Figure 3 outlines the potential mechanistic pathways underlying these major health benefits, which are discussed in detail below.

### 3.1. Antioxidant Activity

Chickpea has been widely shown to possess in vitro antioxidant activity [32], and this capacity mainly derives from its abundant phenolic and flavonoid constituents, which are concentrated in the seed coat, with dark-colored seeds exhibiting 13-fold, 11-fold, and 31-fold higher total polyphenol content, total flavonoid content, and overall antioxidant activity than cream-colored and beige seeds [33]. Phenolic compounds are recognized for their ability to eliminate reactive oxygen species, and they also bind metal ions to mitigate oxidative stress and protect cellular systems, with genistein and biochanin A representing typical antioxidant agents [21].

Processing treatments can further enrich antioxidant components. For instance, solid-state fermentation of chickpea utilizing the *Cordyceps militaris* strain SN-18 significantly elevates the contents of protocatechuic acid, chlorogenic acid, rutin, genistein, daidzein, and biochanin A, thereby enhancing the overall antioxidant capacity [34]. Furthermore, germination treatments can also effectively improve the antioxidant activity of phenolic fractions in chickpea. Regarding specific health benefits, in vivo functional assessments demonstrate that dietary inclusion of chickpea hulls improves antioxidant markers in rats through their polyphenolic components, and modulates the hindgut microecology via their fiber constituents. Concurrently, chickpea extracts exhibit pronounced hepatoprotective effects against carbon tetrachloride-induced liver injury, a health benefit explicitly attributed to the antioxidant and anti-inflammatory activities of their isoflavones and phenolic acids.

### 3.2. Anti-Obesity Effects and Lipid Metabolism

With cardiovascular disease and diabetes presenting persistent global challenges, and in view of the limitations of conventional pharmacotherapies, functional foods rich in plant-derived nutrients offer an appealing strategy for improving lipid and glucose profiles [35]. As a nutrient-dense crop with a low glycemic index (GI ≈ 32–36), chickpea exhibits tremendous potential in ameliorating glucolipid metabolism and combating obesity [36,37]. This is primarily attributed to its abundant soluble dietary fiber, phytosterols, and saponins. Studies indicate that incorporating chickpea into the habitual diet modifies overall fatty acid and fiber intake, thereby contributing to improved serum lipid profiles and better glucose regulation [38].

At the molecular level, soyasaponin βg, a core lipid-lowering constituent, can bind to bile acids and exogenous cholesterol in the intestinal tract, interrupting their enterohepatic circulation. This process compels the liver to accelerate the consumption of endogenous cholesterol for bile acid synthesis, thereby exerting a pronounced hypolipidemic efficacy. Furthermore, the specific isoflavone component biochanin A demonstrates outstanding performance in intervening against metabolic syndrome. It not only exhibits remarkable antioxidant efficacy in diabetic models [39], but also leverages its reducing and metal-chelating properties to suppress lipopolysaccharide (LPS)-induced microglial activation and protect dopaminergic neurons. This affords critical target-organ protection for alleviating neurological complications triggered by glucolipid metabolic disorders.

### 3.3. ACE-Inhibitory Activity

Hypertension represents a major risk factor for stroke and other cardiovascular diseases, and its development is closely associated with ACE activity. ACE catalyzes the conversion of inactive angiotensin I into the potent vasoconstrictor angiotensin II and simultaneously inactivates the vasodilator bradykinin, thereby driving elevations in blood pressure [40]. Chickpea proteins constitute a valuable source of peptides with ACE inhibitory potential.

Long-term consumption of chickpea can effectively reduce serum low-density lipoprotein cholesterol (LDL-C) and triglyceride levels, and exhibits remarkable efficacy in improving vascular endothelial relaxation function and regulating mild-to-moderate hypertension, thereby comprehensively mitigating the pathological risks of atherosclerosis and coronary heart disease. Early work demonstrated that chickpea protein hydrolysates generated through Alcalase digestion contain abundant peptides exhibiting ACE inhibition, with half-maximal inhibitory concentrations (IC50) ranging from 0.11 to 0.21 mg/mL [41]. Subsequent studies showed that the type of protease used during hydrolysis markedly influences the inhibitory potency of the resulting peptides, and that effective inhibitors must withstand gastrointestinal digestion and reach the circulation before entering target cells [42]. Specific short peptide sequences released upon the digestion of chickpea globulin can competitively occupy the active site of ACE, exerting excellent ACE inhibitory activity (analogous to the pharmacological mechanism of antihypertensive drugs), thereby promoting vasodilation.

### 3.4. Anticancer Activity

Cancer remains one of the leading causes of mortality worldwide, and its onset and progression are deeply linked to dietary patterns. Dietary intervention therefore represents an important means of prevention and modulation. In vitro experiments show that chickpea protein hydrolysates suppress the proliferation of several cancer cell lines. They almost completely inhibit the growth of colon cancer Caco-2 cells and display marked antiproliferative activity against breast cancer MDA-MB-231 cells (12–14%) and prostate cancer LNCaP (22–35%) and PC-3 (32–37%) cells, indicating that chickpea-derived peptides hold anticancer potential [26]. Notably, hydrolysates produced by Alcalase and Flavorzyme can partially substitute serum to support the growth of THP-1 cells, though this effect is not observed in Caco-2 cells [43].

Animal studies further confirm that diets containing 2% or 10% cooked chickpea suppress colon carcinogenesis in mice [44]. The protective mechanism may involve enhanced CD8^+^ T-cell activity and improved responsiveness to anti-PD-1 therapy, with microbial butyrate production playing a critical regulatory role [45]. Additionally, lycopene and other components present in chickpea have been associated with reduced prostate cancer risk, providing further support for its anticancer relevance.

### 3.5. Antifungal Activity

In vitro findings indicate that chickpea seed extracts exhibit inhibitory activity against multiple Gram-negative bacteria at concentrations of 16–64 μg/mL, whereas inhibition of Gram-positive organisms occurs at relatively higher concentrations of 64 μg/mL or above [46]. Beyond small-molecule extracts, chickpea seeds themselves encode and produce antifungal peptides. Earlier studies isolated two peptides with novel N-terminal sequences, named cicerin (8.2 kDa) and arietin (5.6 kDa), both of which inhibited *Aspergillus flavus*, *Fusarium oxysporum*, and *Botrytis cinerea*.

The antimicrobial actions of chickpea extracts likely arise from a multitarget mechanism. Similarly to many plant-derived bioactive compounds, its phenolics and flavonoids may act synergistically by disrupting microbial membrane integrity, inhibiting key enzymatic processes, and interfering with intracellular biosynthetic pathways [47]. This multifaceted mode of action reduces the likelihood of resistance development and supports chickpea’s potential as a natural preservative or antimicrobial agent.

### 3.6. Anti-Inflammatory Activity

Chronic inflammation is a common feature across many long-term diseases. A substantial body of evidence indicates that inappropriate activation and expression of the NF-κB is a central driver of inflammatory responses [48]. Phenolic extracts are capable of attenuating NF-κB activation, and chickpea-derived compounds such as biochanin A, its 7-O-β-D-glucoside derivative, and daidzein can downregulate NF-κB, COX-2, and TNF-α, thereby suppressing aluminum chloride-induced neuroinflammation [49].

Studies employing inflammation-activated RAW264.7 cells and nitric oxide assays show that concentrates from germinated chickpea exhibit stronger anti-inflammatory effects than cooked samples, with phenolic constituents identified as the key active components [50]. Additional work further confirms that germination enhances the anti-inflammatory capacity of chickpea [51].

### 3.7. Other Biological Activities

Chickpea also demonstrates analgesic, diuretic, aphrodisiac, and carminative properties, although some of these effects require further validation. Furthermore, it has been reported that anti-nutritional factors in chickpea, such as phytic acid, exhibit potential activities in modulating immune functions and enhancing mineral bioavailability when present at appropriate concentrations [52]. In recent years, growing attention has focused on its ability to lower blood pressure and improve insulin resistance. These actions are thought to arise from delayed glucose absorption mediated by resistant starch and high-amylose fractions, together with the blood pressure regulatory potential of linoleic acid and β-sitosterol [53]. Chickpea also exhibits potential roles in immunomodulation [54], anticonvulsant effects [55], iron chelation [56], and anticancer activity [44], reflecting its broad spectrum of biological functions.

## 4. Abiotic Stress and the Reconstruction of Chickpea Nutritional Quality

**Figure 4:**
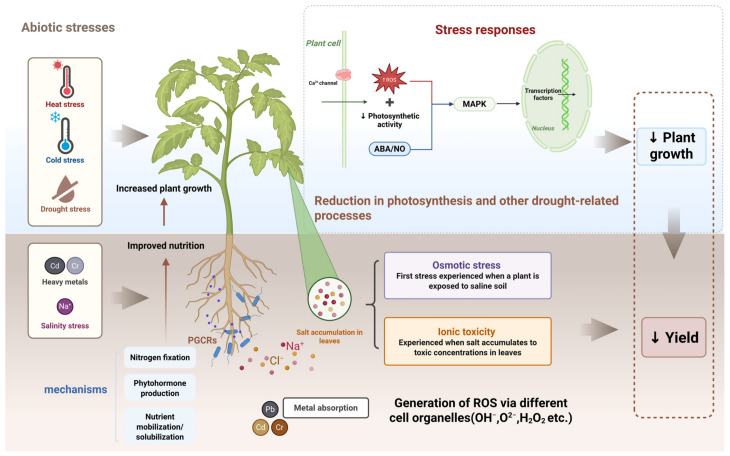
Mechanisms of chickpea response to abiotic stress. Arrows represent: (1) wide grey, flow of stress-induced processes; (2) thin colored, molecular regulatory interactions; and (3) vertical/directional, impacts on plant growth and yield.

Chickpea production is frequently restricted by drought, heat, and salinity, all of which constitute major abiotic stress factors. To cope with these adverse environmental constraints, intricate internal defense networks and metabolic reprogramming mechanisms are activated within the plant system (Figure 4).

### 4.1. Drought Stress and Nutritional Quality Regulation

Drought typically accounts for an estimated 40–50% annual yield loss in global chickpea cultivation, where water deficit triggers internal metabolic reprogramming through escape, avoidance, and tolerance mechanisms [57,58,59]. Untargeted metabolomics indicates that osmotic stress selectively induces the overaccumulation of allantoin, L-proline, and multiple essential amino acids, which not only optimizes the composition of dietary organic nitrogen sources but also confers significant radical-scavenging and immunomodulatory activities on the raw grain [60]. Furthermore, the spatial physicochemical reorganization of starch, protein, and slow-digesting carbohydrates induced by drought effectively lowers the glycemic index of the final food products, while the resulting carbohydrate fractions function as premium prebiotics that efficiently support the proliferation of beneficial microbiota in the intestinal tract [61].

At the secondary metabolism level, the oxidative pressure elicited by water deficit activates the phenylpropanoid pathway, leading to a significant quantitative increase in total polyphenols and total flavonoids within the seed coat and endosperm compared to the control counterparts [62]. Among these, concentration elevations are most prominent for ascorbic acid, a dietary antioxidant, and highly bioactive isoflavone monomers such as biochanin A.

### 4.2. Heat Stress and Lipid Metabolism Reconstruction

Heat stress significantly suppresses chickpea biomass accumulation by compromising photosynthesis, degrading chlorophyll, and destabilizing cellular membrane systems [63,64]. When ambient temperatures persistently exceed 35 °C, reproductive development is most severely impaired because pollen exhibits a profoundly higher sensitivity to thermal stimuli than pistils, culminating in up to a 39% reduction in final yield [65,66]. The accompanying oxidative stress drives an excessive accumulation of reactive oxygen species and triggers severe lipid peroxidation. From a food science perspective, this cascade accelerates the degradation of seed unsaturated fatty acids, deteriorating the fatty acid profile of the raw grain and curtailing the antioxidant shelf life of processed products [67].

Conversely, as a defensive mechanism, thermal adversity activates secondary metabolic pathways, upregulating the synthesis of endogenous phenolics, flavonoids, and heat shock proteins to preserve cellular integrity [68,69]. Based on reproductive indicators such as pollen germination and pod setting, researchers have successfully identified six thermotolerant genotypes and integrated them into cross-breeding programs [70]. Selecting specific mutant lines to optimize the activities of key enzymes in fatty acid synthesis and secondary metabolism effectively suppresses heat-induced lipid rancidity. This targeted breeding approach ensures that chickpeas harvested under thermal stress maintain high enrichment of unsaturated fatty acids, total polyphenols, and flavonoids.

### 4.3. Cold Stress and Membrane Lipid Stability

The optimal diurnal temperature range for the vegetative growth of chickpea is 29/21 °C to 21/15 °C, whereas low temperatures of 3–8 °C severely inhibit seed germination and suppress seedling vigor [71,72]. Chilling-induced pollen sterility and floral organ abortion during the flowering stage constitute the primary factors limiting chickpea yield [73]. Cold stress compromises plasma membrane integrity and transport barriers, macroscopically culminating in growth stagnation and tissue necrosis; notably, the core metabolic divergence between cold-tolerant and cold-sensitive genotypes manifests primarily in the accumulation of dietary antioxidant and osmoregulatory factors, such as ascorbic acid and free proline [74].

As a defense and quality remodeling strategy, chickpea upregulates lipoxygenase activity and increases the double bond index, significantly elevating the proportion of unsaturated fatty acids, such as linoleic acid, in the plasma membrane to maintain membrane fluidity and stability [75,76]. Furthermore, the application of exogenous glycine betaine effectively improves the functionality and biochemical nutrient metabolism of reproductive organs, mitigating cold-induced reproductive failure and providing a feasible pathway to enhance the ultimate nutrient retention of chickpea seeds under environmental adversity.

### 4.4. Salt Stress and Secondary Metabolism Regulation

Soil salinity affects approximately 20% of global irrigated croplands, culminating in an 8–10% annual yield loss in salt-sensitive chickpea [77,78]. Salinity-induced osmotic stress and ionic toxicity restrict root water absorption, trigger nutritional imbalances and premature senescence, and ultimately suppress photosynthesis and carbon assimilation [77,79]. This impaired physiological state of primary metabolism severely hinders the biosynthesis of storage proteins and starch during seed development, consequently deteriorating the processing functionalities and nutritional density of chickpea.

Although vegetative growth is suppressed, elevated salinity functions as a potent extracellular stimulus that vigorously activates the secondary metabolic defense network of chickpea. The introduction of arbuscular mycorrhizal fungi or exogenous proline reinforces endogenous antioxidant enzyme activities, synergistically accelerating the accumulation of health-promoting factors, including total polyphenols and total flavonoids [80]. The intracellular accumulation of specific phenolic fractions substantially enhances the radical-scavenging efficiency of chickpea, thereby conferring vital dietary health benefits—such as mitigating chronic metabolic syndrome and modulating blood glucose levels—upon the final processed products [68].

### 4.5. Other Stress and Food Safety

Heavy metal ions readily accumulate in chickpea seeds via transmembrane transport, directly triggering food safety risks. Oxidative stress and metabolic disorders induced by typical heavy metals, such as chromium and cadmium, significantly suppress the biosynthesis of major storage proteins and starch during the seed development phase, thereby compromising the fundamental nutritional value, pasting properties, and overall processing functionality of chickpea [81,82]. To counteract heavy metal toxicity, the plant activates endogenous detoxification and secondary metabolic networks, driving the substantial accumulation of free proline, phytochelatins, and phenolic fractions within the seeds. Although the accumulation of these components enhances the in vitro antioxidant activity of the raw grain, the severe degradation of primary nutrients and the persistence of exogenous heavy metal residues still pose substantial threats to food processing quality, sensory flavor, and long-term human health [83].

## 5. Chickpea Quality Breeding and By-Product Processing

### 5.1. Co-Selection of Climate Resilience and Functional Components

In response to global nutritional challenges, modern chickpea breeding increasingly focuses on the co-selection of agronomic resilience and nutritional quality. Recent findings indicate that irrigation timing exerts a decisive influence on the nutritional composition of chickpea: irrigation before flowering yields the highest protein content at 29.52%, whereas irrigation at early flowering favors starch accumulation at 36.30% [84]. To broaden the narrow genetic base of cultivated germplasm, wide hybridization incorporating 1476 wild accessions is deployed to introduce elite alleles for environmental adaptation [85]. Concurrently, genome-wide association studies (GWAS) are utilized to dissect the genetic architecture governing fatty acid profiles and micronutrient configurations, thereby guiding the targeted biofortification of core minerals such as iron and zinc [86].

Building upon these genomic insights, the integration of marker-assisted selection (MAS) enables the precise anchoring of quantitative trait loci (QTLs) linked to drought [87], extreme temperature [88,89], salinity [90], and heavy metal tolerance. Currently, utilizing specific allelic variants of the *CaStGR1* gene permits the efficient enrichment of carotenoids within seeds while fully preserving environmental plasticity [91]. In parallel, multiplex CRISPR-Cas9 genome editing facilitates the targeted knockout of specific lipoxygenase (*CaLOX*) genes to effectively suppress stress-induced lipid rancidity; meanwhile, modulating elite transcription factors, including *CaMyb* and *CaDREB*, synchronously activates osmotic defense responses and accelerates the biosynthesis of health-promoting isoflavones like biochanin A [9,57]. Although gene-editing technologies demonstrate remarkable improvement efficacy, research on transgenic chickpea remains relatively limited. This regulatory alignment successfully transforms environmental adversity into upstream physiological stimuli, ultimately securing the directional selection of high-yielding, nutrient-dense, and stable ideotypes with optimized quality profiles.

### 5.2. By-Product Valorization and Safety Control

Globally, chickpea utilization is expanding from traditional preparations such as hummus and soups toward modern industrial products, including flour, functional snacks, plant-based beverages, and coffee substitutes [92,93]. The specific forms of chickpea utilization within food processing sectors are summarized in Table 2.

However, the concentration of antinutritional factors—such as tannins, saponins, and phytic acid—along with nonspecific lipid-transfer proteins and α-amylase inhibitors in raw materials can provoke allergic reactions [94,95]. Proteomic screening has identified seven putative cross-reactive IgE-mediated allergens, severely constraining their high-value dietary applications [96]. The deactivation efficiency of these hazardous factors is tightly coupled with the physicochemical functionality of the by-product matrices: although conventional pressure cooking can eliminate up to 93.97% of tannins and 87.71% of total polyphenols to neutralize primary antinutritional constraints, intense thermal shearing often compromises the physicochemical and processing functionalities of macromolecular proteins within the by-products [97]. Conversely, controlled biological transformations such as germination and solid-state fermentation not only significantly enhance the in vitro digestibility of starch and protein within the by-products but also selectively cleave the linear IgE-binding epitopes of allergenic proteins through advanced multi-modal degradation networks; furthermore, integration with instant controlled pressure-drop (DIC) technology or controlled Maillard reaction-based modifications can profoundly disrupt the tertiary spatial conformation of potential allergens [30,98,99]. When combined with pressure cooking, these strategies enhance food safety by simultaneously reducing antinutritional factors and minimizing allergenic risks. Future research should focus on optimizing and integrating multi-method synergistic modification technologies to systematically construct immunological and toxicological safety control systems for deeply processed chickpea by-products, thereby thoroughly eliminating hazardous components while maximizing the preservation of their nutritional configurations to promote their widespread application in functional dietary systems.

**Table 2:** Applications of chickpeas in food processing.

Types	Processing	Product	References
Desi	Roasted	Channa dal	[100]
		Hummus	
		Dhal	[101]
Kabuli	Roasted, boiled	Salad, Vegetable mixes	[102]
		Stews, Soups	[101]
Chickpea husks	Extraction	Baking Additives	[101]
Chickpea	Milling	Chickpea flour	[18]
	Cooking, canning	Aquafaba	
	Roasted	Leblebi	[103]
	Roasted	Sattu	[3]
	Shaping	Chilla	
	Fried	Boondi	[102]
	Steamed	Dhokla	
	Roasted	Pasta	[101]
	Milling	Chickpea coffee	[104]
	Baking	Supplement cookies	[101]
	Extraction	Facial masks	[105]
		Confectionery	[105]
Chickpea hulls	Extraction	Textile dye	[18]
Chickpea straw	Drying, Pelletizing	Animal feed	[18]
Aquafaba	Cooking	Foams and Emulsions	[18]

## 6. Conclusions and Perspectives

Chickpea, as a nutrient-dense functional food, holds significant potential for advancing human health and supporting sustainable agriculture. This review synthesizes its multifaceted value and future prospects, emphasizing its rich composition of high-quality protein, dietary fiber, unsaturated fatty acids, and a wide range of bioactive compounds that collectively contribute to antioxidant, anti-inflammatory, hypoglycemic, hypolipidemic, and anticancer functions through coordinated regulation of multiple signaling pathways. However, production is severely constrained by drought, heat, and salinity. Concurrently, the triggered metabolic reprogramming induces the hyper-accumulation of functional secondary metabolites, including phenolic compounds. Consequently, modern breeding strategies integrating multi-omics and genome-editing technologies have shifted their focus toward the directional reconstruction of specific functional components, thereby accelerating the development of high-yielding, nutrient-dense, and stress-tolerant cultivars. When coupled with high-value valorization processing such as fermentation and enzymatic hydrolysis, these paradigms successfully establish a synergistic pathway linking environmental adversity to consumer health benefits. Future research should prioritize the synergistic regulatory mechanisms governing nutritional quality and plant defense networks under concurrent field abiotic stresses, systematically enhancing the core value of chickpea within sustainable functional food systems.

## Figures and Tables

**Table 1:** Nutrient compositions of chickpeas.

Type	Grouping	Nutrients	Reported Values					
			Literature Values	Genotypes		Processing Methods		
				Kabuli	Desi	Raw	Soaked	Cooked
Protein	Total Content	Total protein (g/100 g dw)	18.3–25.2 ^b^	24.51 ± 0.27 ^d^	20.29 ± 0.13 ^d^	28.33 ± 0.31 ^f^	26.27 ± 1.94 ^f^	19.87 ± 0.12 ^f^
	Amino Acids	Glutamic acid (g/100 g protein)	18.7 ± 0.1 ^a^	16.71 ± 0.09 ^d^	14.90 ± 0.06 ^d^	NR	NR	NR
		Aspartic acid (g/100 g protein)	13.5 ± 0.1 ^a^	11.66 ^e^	10.59 ^e^	NR	NR	NR
		Arginine (g/100 g protein)	10.4 ± 0.1 ^a^	8.07 ^e^	8.11 ^e^	NR	NR	NR
		Leucine (g/100 g protein)	8.0 ± 0.1 ^a^	2.48 ± 0.02 ^d^	4.24 ± 0.04 ^d^	NR	NR	NR
		Lysine (g/100 g protein)	7.2 ± 0.1 ^a^	5.47 ^e^	5.55 ^e^	NR	NR	NR
		Phenylalanine (g/100 g protein)	6.0 ± 0.1 ^a^	5.81 ^e^	5.42 ^e^	NR	NR	NR
		Alanine (g/100 g protein)	4.6 ± 0.0 ^a^	3.52 ± 0.02 ^d^	4.11 ± 0.04 ^d^	NR	NR	NR
		Valine (g/100 g protein)	4.1 ± 0.0 ^a^	3.20 ± 0.04 ^d^	4.69 ± 0.08 ^d^	NR	NR	NR
		Serine (g/100 g protein)	5.9 ± 0.1 ^a^	7.33 ± 0.03 ^d^	5.40 ± 0.03 ^d^	NR	NR	NR
		Isoleucine (g/100 g protein)	3.6 ± 0.1 ^a^	3.90 ^e^	3.70 ^e^	NR	NR	NR
		Glycine (g/100 g protein)	4.3 ± 0.1 ^a^	2.54 ^e^	3.12 ^e^	NR	NR	NR
		Threonine (g/100 g protein)	4.1 ± 0.0 ^a^	3.13 ^e^	3.23 ^e^	NR	NR	NR
		Tyrosine (g/100 g protein)	2.3 ± 0.0 ^a^	6.93 ± 0.05 ^d^	2.87 ± 0.02 ^d^	NR	NR	NR
		Histidine (g/100 g protein)	2.3 ± 0.1 ^a^	2.70 ± 0.03 ^d^	3.27 ± 0.05 ^d^	NR	NR	NR
		Proline (g/100 g protein)	1.8 ± 0.1 ^a^	2.95 ± 0.01 ^d^	3.63 ± 0.02 ^d^	NR	NR	NR
		Tryptophan (g/100 g protein)	0.8 ± 0.0 ^a^	NR	NR	NR	NR	NR
		Methionine (g/100 g protein)	0.8 ± 0.0 ^a^	1.92 ^e^	2.05 ^e^	NR	NR	NR
		Cystine (g/100 g protein)	1.1 ± 0.1 ^a^	0.19 ^e^	0.15 ^e^	NR	NR	NR
Carbohydrates	Total Content	Total carbohydrates (g/100 g dw)	34.20–54.72 ^c^	NR	NR	64.07 ± 0.28 ^f^	64.42 ± 1.90 ^f^	70.44 ± 0.34 ^f^
	Polysaccharides	Total starch (g/100 g dw)	36.91 ± 0.60 ^c^	NR	NR	NR	NR	NR
	Sugars (Mono/Di)	Total sugars (g/100 g dw)	10.8 ^c^	NR	NR	9.10 ± 0.00 ^f^	8.41 ± 0.12 ^f^	6.22 ± 0.07 ^f^
		Fructose (g/100 g dw)	1.89 ± 0.08 ^c^	NR	NR	0.84 ± 0.06 ^f^	0.71 ± 0.05 ^f^	0.57 ± 0.06 ^f^
		Sucrose (g/100 g dw)	NR	3.10–4.41 ^e^	1.56–2.85 ^e^	3.25 ± 0.07 ^f^	3.03 ± 0.00 ^f^	2.02 ± 0.03 ^f^
		Trehalose (g/100 g dw)	NR	NR	NR	0.46 ± 0.03 ^f^	0.44 ± 0.03 ^f^	0.27 ± 0.01 ^f^
	Oligosaccharides	Raffinose (g/100 g dw)	1.45 ± 0.07 ^c^	0.48–0.73 ^e^	0.46–0.77 ^e^	0.93 ± 0.05 ^f^	0.76 ± 0.02 ^f^	0.55 ± 0.07 ^f^
		Stachyose (g/100 g dw)	2.56 ± 0.08 ^c^	1.76–2.72 ^e^	1.25–1.98 ^e^	2.29 ± 0.03 ^f^	2.25 ± 0.02 ^f^	1.85 ± 0.02 ^f^
		Verbascose (g/100 g dw)	0.19 ± 0.06 ^c^	NR	NR	1.34 ± 0.02 ^f^	1.22 ± 0.01 ^f^	0.97 ± 0.01 ^f^
Dietary fiber	Total Content	Total dietary fiber (g/100 g dw)	3.82 ± 0.13 ^c^	21.86 ± 0.55 ^d^	18.73 ± 0.52 ^d^	12.2 ^g^	NR	7.6 ^g^
	Fiber Fractions	Soluble fiber (g/100 g dw)	1.23–1.38 ^b^	NR	NR	NR	NR	NR
		Insoluble fiber (g/100 g dw)	14.1–23.2 ^b^	NR	NR	NR	NR	NR
Lipids	Total Content	Total lipids (g/100 g dw)	1.12–6.80 ^b^	NR	NR	NR	NR	NR
		Fat (g/100 g dw)	6.48 ± 0.08 ^c^	5.20 ± 0.87 ^d^	6.54 ± 0.44 ^d^	4.31 ± 0.09 ^f^	6.15 ±0.21 ^f^	7.22 ± 0.17 ^f^
	Saturated FA	Saturated fatty acid (% total fatty acid)	NR	NR	NR	12.41 ± 0.54 ^f^	11.77 ± 0.04 ^f^	12.23 ± 0.00 ^f^
		Palmitic (C16:0) (% total fatty acid)	NR	9.41 ^e^	9.09 ^e^	NR	NR	NR
		Stearic (C18:0) (% total fatty acid)	NR	1.42 ^e^	1.16 ^e^	NR	NR	NR
		Arachidic (C20:0) (% total fatty acid)	NR	2.69 ^e^	3.15 ^e^	NR	NR	NR
	Monounsaturated FA	Monounsaturated fatty acid (% total fatty acid)	NR	NR	NR	25.18 ± 0.81 ^f^	24.10 ± 0.76 ^f^	24.62 ± 0.00 ^f^
		Palmitoleic (C16:1) (% total fatty acid)	NR	0.30 ^e^	0.26 ^e^	NR	NR	NR
		Oleic (C18:1) (% total fatty acid)	NR	32.56 ^e^	22.31 ^e^	NR	NR	NR
		Polyunsaturated fatty acid (% total fatty acid)	NR	NR	NR	62.41 ± 0.27 ^f^	64.13 ± 0.80 ^f^	63.14 ± 0.00 ^f^
		Linoleic (C18:3) (% total fatty acid)	NR	51.20 ^e^	61.62 ^e^	NR	NR	NR
	Fatty Acid Ratios	Oleic acid/linoleic acid (% total fatty acid)	NR	0.66 ^e^	0.51 ^e^	NR	NR	NR
Minerals	Total Content	Total minerals (mg/100 g dw)	NR	NR	NR	NR	NR	NR
	Macro-elements	Calcium (mg/100 g dw)	176 ^c^	187.25 ± 3.32 ^d^	177.94 ± 3.42 ^d^	57 ^g^	NR	49 ^g^
		Magnesium (mg/100 g dw)	176 ^c^	3.88 ± 0.08 ^d^	3.71 ± 0.5 ^d^	79 ^g^	NR	48 ^g^
		Phosphorus (mg/100 g dw)	226 ^c^	394.0 ^e^	451.5 ^e^	252 ^g^	NR	168 ^g^
		Potassium (mg/100 g dw)	870 ^c^	1060.0 ^e^	994.5 ^e^	718 ^g^	NR	291 ^g^
		Sodium (mg/100 g dw)	121 ^c^	11.26 ± 1.44 ^d^	7.35 ± 0.65 ^d^	24 ^g^	NR	7 ^g^
	Trace Elements	Zinc (mg/100 g dw)	4.32 ^c^	4.18 ± 0.23 ^d^	3.32 ± 0.27 ^d^	2.76 ^g^	NR	1.53 ^g^
		Copper (mg/100 g dw)	1.10 ^c^	0.70 ^e^	0.58 ^e^	0.656 ^g^	NR	0.352 ^g^
		Manganese (mg/100 g dw)	2.11 ^c^	115.53 ± 2.61 ^d^	133.63 ± 1.85 ^d^	21.306 ^g^	NR	1.030 ^g^
		Iron (mg/100 g dw)	7.72 ^c^	51.11 ± 3.74 ^d^	48.26 ± 2.47 ^d^	4.31 ^g^	NR	2.89 ^g^
Vitamins	Total Content	Total vitamins (mg/100 g dw)	NR	NR	NR	NR	NR	NR
	Water- soluble	Vitamin C (mg/100 g dw)	1.3 ^c^	1.34 ^e^	1.65 ^e^	4.0 ^g^	NR	1.3 ^g^
		Thiamin (B1) (mg/100 g dw)	0.453 ± 0.007 ^c^	0.49 ^e^	0.29 ^e^	0.477 ^g^	NR	0.116 ^g^
		Riboflavin (B2) (mg/100 g dw)	0.173 ± 0.007 ^c^	0.26 ^e^	0.21 ^e^	0.212 ^g^	NR	0.063 ^g^
		Niacin (B3) (mg/100 g dw)	1.603 ± 0.012 ^c^	1.22 ^e^	1.72 ^e^	1.541 ^g^	NR	0.526 ^g^
		Pantothenic acid (B5) (mg/100 g dw)	0.286 ^c^	1.02 ^e^	1.09 ^e^	1.588 ^g^	NR	0.286 ^g^
		Pyridoxine (mg/100 g dw)	0.466 ± 0.008 ^c^	0.38 ^e^	0.30 ^e^	NR	NR	NR
		Vitamin B6 (mg/100 g dw)	0.139 ^c^	NR	NR	0.535 ^g^	NR	0.139 ^g^
		Folate (mg/100 g dw)	0.172 ^c^	299.0 ^e^	206.5 ^e^	557 ^g^	NR	172 ^g^
		Choline (mg/100 g dw)	NR	NR	NR	99.3 ^g^	NR	42.8 ^g^
	Fat-soluble & Carotenoids	Vitamin A (mg/100 g dw)	0.016 ^c^	NR	NR	67 ^g^	NR	27 ^g^
		Vitamin K (mg/100 g dw)	NR	NR	NR	9.0 ^g^	NR	4.0 ^g^
		Vitamin E (mg/100 g dw)	0.35 ^c^	NR	NR	0.82 ^g^	NR	0.35 ^g^
		Total carotenoids (mg/100 g β-Carotene)	NR	2.14 ^e^	3.72 ^e^	NR	NR	NR
Phenolic compounds	Total Polyphenols	Total phenolic (mg/100 g dw)	NR	115.6 ^e^	274.0 ^e^	NR	NR	NR
		Flavonoid (mg/100 g dw)	NR	31.39 ^e^	62.25 ^e^	NR	NR	NR
	Isoflavones	Formononetin (% total isoflavone)	2.61–16.6 ^b^	NR	NR	NR	NR	NR
		Biochanin A (% total isoflavone)	17.8–30.0 ^b^	NR	NR	NR	NR	NR
		Biochanin glucoside (% total isoflavone)	13.3–29.1 ^b^	NR	NR	NR	NR	NR
		Daidzein (% total isoflavone)	0.30–20.0 ^b^	NR	NR	NR	NR	NR
		Genistein (% total isoflavone)	10.0–25.5 ^b^	NR	NR	NR	NR	NR
	Tocopherols	Total tocopherols (mg/100 g dw)	NR	NR	NR	11.71 ± 0.76 ^f^	18.28 ± 0.22 ^f^	22.20 ± 0.52 ^f^
		α-Tocopherol (mg/100 g dw)	NR	NR	NR	2.36 ± 0.16 ^f^	3.72 ± 0.02 ^f^	4.42 ± 0.20 ^f^
		β-Tocopherol (mg/100 g dw)	NR	NR	NR	0.07 ± 0.00 ^f^	0.10 ± 0.01 ^f^	0.13 ± 0.01 ^f^
		γ-Tocopherol (mg/100 g dw)	NR	NR	NR	8.52 ± 0.53 ^f^	13.45 ± 0.25 ^f^	16.44 ± 0.35 ^f^
		δ-Tocopherol (mg/100 g dw)	NR	NR	NR	0.76 ± 0.06 ^f^	1.00 ± 0.01 ^f^	1.21 ± 0.04 ^f^

Note: dw = dry weight; NR = not reported. The superscripts (a–g) denote the corresponding bibliographic references from which the data were compiled: a = [12], b = [13], c = [14], d = [15], e = [2], f = [16], and g = [17].

## Data Availability

No new data were created or analyzed in this study. Data sharing is not applicable to this article.
